# Delayed Lithium Reintoxication in a Case of Severe Multidrug Intoxication: A Case Study

**DOI:** 10.1097/FTD.0000000000001099

**Published:** 2023-08-21

**Authors:** Jiayi Liang, Christiaan J. van den Bout, Tessa M. Bosch, Lieke Mitrov-Winkelmolen

**Affiliations:** * Department of Hospital Pharmacy, Maasstad Hospital, Rotterdam, the Netherlands;; † Department of Hospital Pharmacy, Erasmus University Medical Center, Rotterdam, the Netherlands;; ‡ Department of Intensive Care Unit, Maasstad Hospital, Rotterdam, the Netherlands; and; § MaasstadLab Clinical Pharmacology and Toxicology, Maasstad Hospital, Rotterdam, the Netherlands.

**Keywords:** lithium, intoxication, clearance-inducing therapy, intensive care unit

## Abstract

The authors present a case of severe multidrug intoxication following massive ingestion of lithium, nortriptyline, aripiprazole, lorazepam, and temazepam. After initial treatment, serum lithium levels decreased significantly. However, 28 hours post ingestion, recurrent elevated lithium levels were observed, and serum lithium level increased 0.71 mmol/L in 12 hours. The intensivist consulted a hospital pharmacist about this. After administering clearance-inducing therapy using continuous venovenous hemodialysis, the lithium level was reduced to a long-lasting nontoxic level. The occurrence of secondary elevation in lithium levels exceeding the toxic limit in cases of massive ingestion of lithium tablets, whether in combination with anticholinergic drugs, should be anticipated. Close monitoring and prompt initiation of clearance-inducing therapy can improve clinical outcomes.

## CLINICIAN

A 48-year-old woman with a history of multiple psychiatric disorders was hospitalized after attempting suicide by consuming various drugs. Approximately 1.5 hours after ingesting 21*g* lithium, 630 mg aripiprazole, 3675 mg nortriptyline, 21 mg lorazepam, and 410 mg temazepam, the patient was brought to hospital A. Upon presentation to the emergency department, the patient vomited several times, with a respiratory rate of 20/min, blood pressure of 108/64 mm Hg, heart rate of 89/min, and temperature of 36.1°C. Cardiac monitoring showed a prolonged QRS (136 milliseconds) and a normal QTc (402 milliseconds). Owing to increased somnolence, the patient was intubated. Hyperhydration with saline, one of the mainstays of lithium poisoning treatment, was initiated. This was followed by gastric lavage and the administration of activated charcoal with laxatives through a nasogastric tube. Although activated charcoal does not adsorb lithium, it was administered due to the coingestion of potentially fatal doses of tricyclic antidepressants and atypical antipsychotic agents. Treatment with sodium bicarbonate was initiated because of the prolonged QRS, which was most likely caused by nortriptyline intoxication. After stabilization, the patient was transferred to the intensive care unit of hospital B for further cardiac and respiratory monitoring. The serum lithium levels were closely monitored. The initial lithium level in A was 2.99 mmol/L. Upon arrival in B, a significant decline in the lithium level was observed (2.28 mmol/L over 4 hours). The patient was normonatremic and had adequate diuresis. The following morning, lithium testing revealed a lithium level of 2.09 mmol/L. Consequently, the half-life increased from 10 hours to 60 hours. At t = 28 hours post ingestion, the drug level steadily rose from 2.09 mmol/L to 2.80 mmol/L. Despite hyperhydration, extensive diuresis, and normal-to-hypernatremia, the lithium clearance was inadequate. Is clearance-inducing therapy recommended at this point?

## TOXICOLOGY CONSULTANT

According to Dutch lithium intoxication guidelines, the toxic limit of lithium is 1.5 mmol/L. In case of chronic intoxication, symptoms may present at a lower threshold.^1,2^ Clearance-inducing therapy should be considered at levels >2.5 mmol/L depending on the clinical presentation.^1^ Under normal conditions, Cmax is reached in 1.5–2 hours after ingestion of the immediate-release formulation. In case of an overdose, Tmax may be delayed by up to 72 hours^3^ This was a case of acute lithium intoxication in addition to chronic lithium use. The associated symptoms and risks of such intoxication are serious owing to the existing distribution in the brain tissue, whereas serum and tissue concentrations have already reached equilibrium. Additional exposure to high levels of lithium can result in severe neurological symptoms, including loss of consciousness, seizures, and coma.^4^ As the serum lithium level exceeded the toxic limit 28 hours after ingestion, any additional delay in elimination would increase neurotoxicity. Moreover, due to sedation with propofol and remifentanil, a neurological assessment was hindered.

Continuous venovenous hemodialysis (CVVHD) was initiated, and the lithium levels were closely monitored to achieve continuous elimination. After 8.5 and 17 hours of elimination acceleration, the lithium level decreased to 1.55 and 1.06 mmol/L, respectively. Continuation of CVVHD for up to 24 hours was advised.

## CLINICIAN

What caused the recurrent elevated lithium level at t = 28 hours?

## TOXICOLOGY CONSULTANT

**FIGURE 1.**
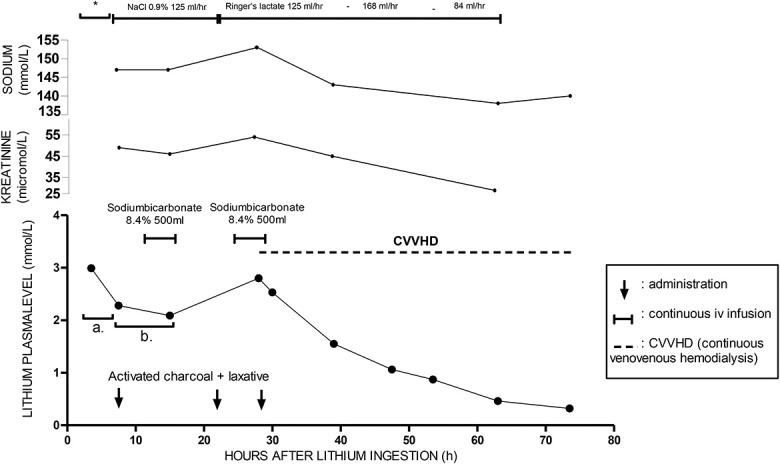
Lithium drug level and applied interventions over time. *Initial treatment: hyperhydration with NaCl 0.9%, sodium bicarbonate 8.4% 300mL, gastric lavage and activated charcoal + laxative. A, the first drop of the lithium serum level, and (B) the second drop of the lithium serum level.

This phenomenon can be explained by the combined effect of 3 mechanisms:

First, the pattern of lithium levels reflects acute-on-chronic lithium intoxication. This was illustrated by the diminished drop between the second and third serum levels compared with the steep drop between the first and second levels (Fig. 1A). The high serum lithium concentration at presentation was the result of acute intoxication with relatively rapid clearance under adequate diuresis and hyperhydration conditions, resulting in direct dilution of the serum lithium concentration. However, the diminished drop in serum lithium levels between the second and third blood samples corresponds to chronic intoxication in which redistribution of intracellular lithium to the extracellular compartment occurs. High intracellular concentrations are observed in chronic lithium users, and redistribution from the intracellular compartment can lead to a significant increase in the serum concentration. This form of redistribution can lead to persistent increases in serum levels. This could be a plausible explanation for the rapid decline in serum concentration in the first few hours after intoxication and the deceleration of the decline between t = 7.5 and 15 hours (Fig. 1B).

Second, the observed increase in the serum lithium concentration at t = 28 hours was unusual because it occurred despite extensive diuresis and hyperhydration. A possible explanation is a formation of a bezoar continuously leaking lithium, as the formation of a bezoar with lithium has been mentioned in previous cases.^5–7^ The massive intake of approximately 270 tablets, along with the poorly soluble lithium carbonate salts, may have contributed to bezoar formation.^8^ Moreover, the patient did not defecate until 30 hours after ingestion, indicating the possibility of ongoing lithium absorption that may not have passed through the gastrointestinal tract.

A third factor that may have contributed to the renewed increase in serum lithium concentration is the reactivation of gastrointestinal motility, which was diminished before by the overdose of nortriptyline (ie, anticholinergic effect), after the administration of activated charcoal and laxatives.^6,7^ This may have increased the absorption of lithium from the formed bezoars.

## CLINICIAN

After a 38-hour session, CVVHD was terminated. By that time, the lithium level reduced to 0.46 mmol/L. Follow-up testing at t = 73.5 hours showed a further decrease; the lithium level was 0.32 mmol/L. The patient was somatically cleared for discharge from the intensive care unit after 5 days.

## CONCLUSION

Massive ingestion of lithium tablets can be complicated by a recurrent elevation in serum lithium levels after an initial adequate response to therapy. A possible explanation is a bezoar leaking lithium over a prolonged period. Abdominal CT could confirm this hypothesis. Reactivating intestinal motility by eliminating anticholinergic side effects may contribute to increased lithium absorption. To effectively reduce the intestinal absorption of lithium, total intestinal lavage should be considered, especially in the case of coingestion with other highly toxic drugs.^9^ However, this requires caution in light of the potential fluid and electrolyte imbalance, which further complicates the course and severity of intoxication. Finally, we suspected that the patient's chronic lithium use resulted in the redistribution of lithium from the intracellular compartment to the extracellular compartment, leading to a dramatically prolonged elimination half-life.

In conclusion, we recommend close monitoring of serum lithium levels in patients with an initial adequate response to therapy. This case report describes a secondary elevation in serum lithium levels 28 hours after ingestion despite extensive diuresis. Therefore, the prompt initiation of dialysis is necessary.
